# iRFP Is a Real Time Marker for Transformation Based Assays in High Content Screening

**DOI:** 10.1371/journal.pone.0098399

**Published:** 2014-06-02

**Authors:** Viola Paulus-Hock, Eric C. Cheung, Patricia Roxburgh, Karen H. Vousden, Andreas K. Hock

**Affiliations:** 1 Cancer Research UK Beatson Institute, Glasgow, United Kingdom; 2 The Institute of Cancer Sciences, University of Glasgow, Glasgow, United Kingdom; Case Western Reserve University, United States of America

## Abstract

Anchorage independent growth is one of the hallmarks of oncogenic transformation. Here we show that infrared fluorescent protein (iRFP) based assays allow accurate and unbiased determination of colony formation and anchorage independent growth over time. This protocol is particularly compatible with high throughput systems, in contrast to traditional methods which are often labor-intensive, subjective to bias and do not allow further analysis using the same cells. Transformation in a single layer soft agar assay could be documented as early as 2 to 3 days in a 96 well format, which can be easily combined with standard transfection, infection and compound screening setups to allow for high throughput screening to identify therapeutic targets.

## Introduction

Anchorage independent growth and focus formation are long established assays of oncogenic transformation [1], [2]. Manual counting of colonies is the standard technique to determine oncogenic growth, although this method is time consuming and prone to subjective bias. To increase throughput as well as to overcome potential subjectivity, protocols to fix and/or stain the cells (e.g. PFA fixation and crystal violet stain, MTT [3], or alamarBlue stain [4]), followed by scanning and quantification with ImageJ or other proprietary software have been developed. This decreases processing time and largely eliminates the risk of human error, but does not document the onset of induced clonal outgrowth and in the case of soft agar the growth dynamic of individual colonies. In addition, the fixation and staining process renders the sample unsuitable for subsequent analysis such as western blotting or qRT-PCR.

Quantification of infrared fluorescent proteins (iRFPs) signal has been shown to be an objective and sensitive marker to determine the proliferation behavior in several systems [5]-[7]. Here we demonstrate that quantification of iRFP is an unbiased and expeditious method to assess focus formation and soft agar growth over time.

## Materials and Methods

### Plasmids / Cell Lines

All plasmids and cell lines used were described previously [7].

### Tissue Culture

iRFP 3T3 (mouse fibroblasts, selected with 4 µg/ml puromycin, Life Technologies, A11138-03) were cultured in 10% DCS (Donor Calf Serum, Life Technologies, 16030–074) supplemented with glutamine (Life Technologies, 25030–081) and were incubated with nutlin (Sigma Aldrich, N6287) and actinomycin D (Sigma Aldrich, A9415) in a 37°C incubator at 5% CO_2_ in 8 well plates (NUNC, 167064) or 96 well plates (Corning 3340) as described [7]. Transfections were carried out using Genejuice (Merck Millipore, 70967) according to manufacturer's instructions. Infections were carried out using the Phoenix eco retroviral system [8].

### Soft Agar Assay

Two layer soft agar assays were carried out in 8 well plates by first pouring a bottom layer of 1 ml 0.75% agarose (Melford, MB1200) in 1X DMEM (from 2X DMEM stock, Millipore, SLM-202-B). After solidification the indicated cell lines (transformed or parental iRFP 3T3 fibroblasts, 2000 cells per well) were mixed with 1 ml of 1X DMEM (from 2X DMEM stock, Millipore, SLM-202-B) with 10% DCS and 0.75% agarose (Melford, MB1200, 1 ml per well), cooled to 41°C and plated on top of the bottom layer. Wells were topped up with 2 ml of growth medium (10% DCS DMEM), which was changed every 2 to 3 days.

Single layer soft agar assays were carried out in 96 well plates by pouring 50 µl 1.5% agar with a final concentration of 1X DMEM (bought as 2X DMEM, see above) into each well. After solidification, cells were plated at the indicated concentrations in 150 µl of growth medium. Quantifications were carried out using LI-COR Odyssey (LI-COR) and Image Studio software (version 2.1.10). Data was plotted using Prism (Graph Pad). Cell numbers were counted using a CASY cell counter (Roche).

## Results and Discussion

**Figure 1:**
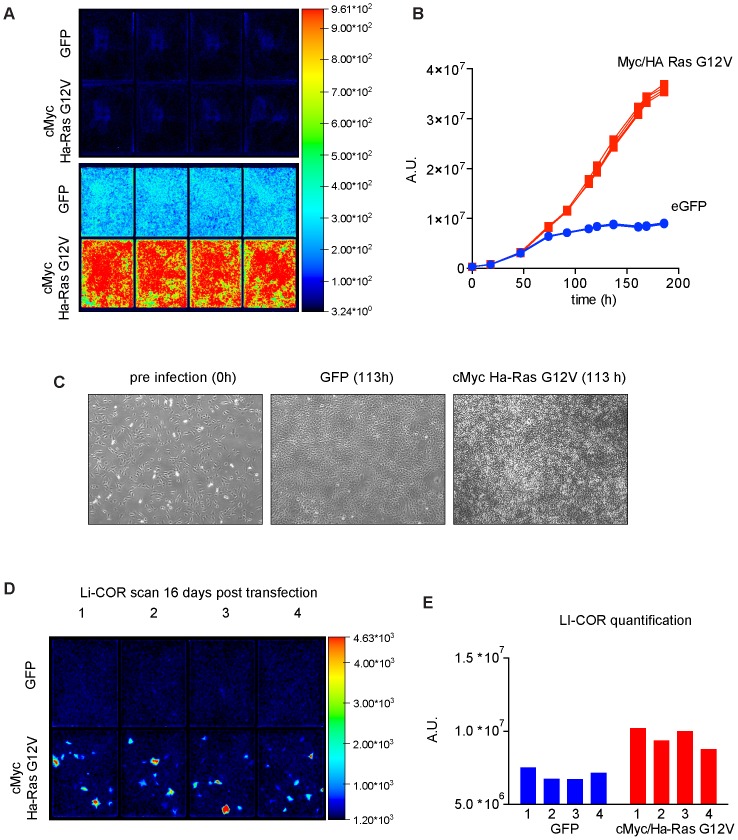
Colony formation analysis via iRFP quantification. (**A**) Odyssey LI-COR scans of cMyc/Ha-RasG12V or eGFP infected iRFP 3T3 fibroblasts at the indicated time points. Scan settings: Resolution 169 µm, offset 2.5 mm, intensity (700 nm) L02. (**B**) Quantification of LI-COR scans at the indicated days. Graph represents the individual signal of replicate wells. Scan settings as in (**A**). (**C**) Light microscopy images before and after infection with the indicated constructs. (**D**) Odyssey LI-COR scans of iRFP 3T3 fibroblasts 16 days post transfection with cMyc/Ha-RasG12V transfected or parental iRFP 3T3 fibroblasts. (**E**) Quantification of LI-COR scans shown in (**D**). Each bar represents the quantification of a single well. Scan settings as in (**A**).

Since we have demonstrated previously that iRFP fluorescence closely correlates with cell number [7], we reasoned that it could also be used to assess different parameters of oncogenic transformation. To test whether iRFP detection can be used to monitor and quantify oncogenic transformation in a 2D focus formation assay (measuring loss of contact inhibition and clonal outgrowth due to loss of anchorage dependence), we plated 3×10^5^ iRFP expressing 3T3 mouse fibroblasts and infected them subsequently with cMyc/Ha-RasG12V or eGFP as a control in 8 well dishes. The plates were scanned repeatedly and the iRFP signal was quantified as a proxy for density. As expected [9], 3T3 cells infected with eGFP grew to confluence and density-arrested subsequently (Figure 1A), which resulted in a plateauing of the fluorescence signal over time after 93 hours (Figure 1B). As shown previously, this demonstrates that once arrested, the control cells do not continue to accumulate iRFP signal. cMyc/Ha-RasG12V infected 3T3 cells initially grew in a similar manner to control cells, but continued to proliferate after 100 hours (Figure 1A, B) and showed a strong increase of iRFP signal compared to untransformed cells (Figure 1B). We did not observe outgrowth of individual colonies, but rather a continuous growth of the great majority of all cMyc/Ha-RAS G12V infected cells (Figure 1C). This is likely due to the high retroviral infection and integration rate [8], which resulted in transformation and loss of contact inhibition of most if not all the cells. To test if iRFP quantification is suitable to determine transformation that happens at a much lower and slower rate, we expressed the same oncogenes in iRFP 3T3 cells by transient transfection. This greatly reduces the number of cells expressing both oncogenes as well as the likelihood of integration and therefore continuation of expression. After 16 days, cMyc/Ha-RasG12V transfected cells showed clear outgrowth of colonies by comparing the parental GFP transfected cells (Figure 1D) and iRFP quantification allowed us to quantify the oncogenic outgrowth per well by an increase of iRFP signal in the oncogene transfected cells (Figure 1E). This demonstrates that iRFP scanning can be used to document the shape and size of colonies without any fixing, staining or other outside interference. In addition, quantification per well is a suitable and simple readout to assess colony-formation assays in an unbiased way without the need for manually counting colonies.

**Figure 2:**
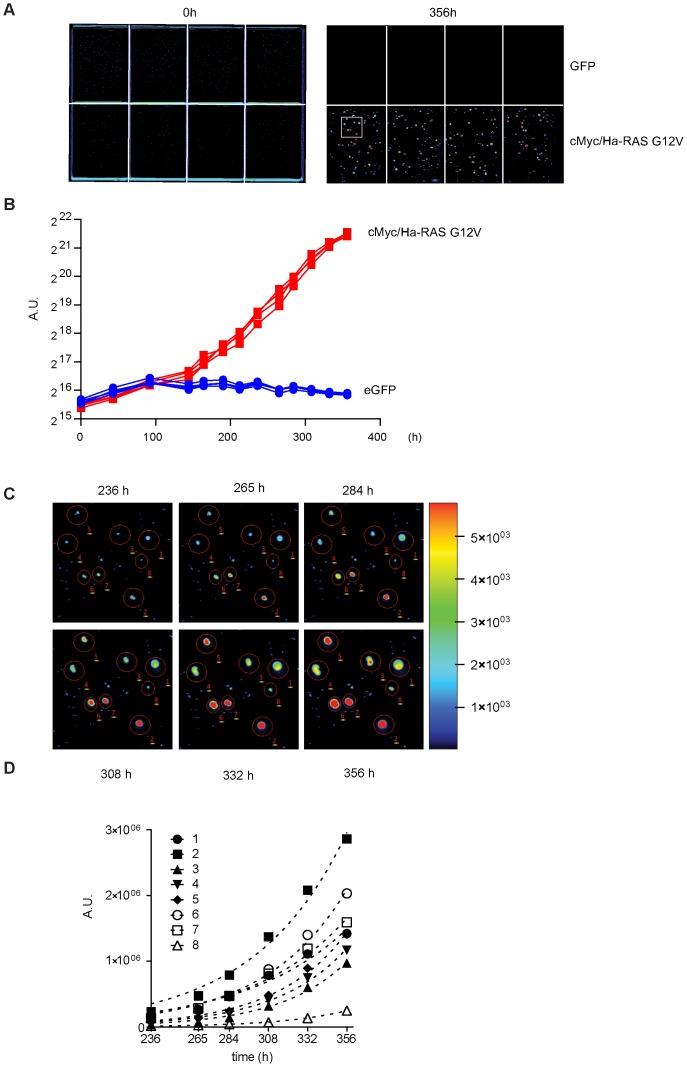
Soft agar assay analysis via iRFP quantification. (**A**) Odyssey LI-COR scans of cMyc/Ha-RasG12V infected or parental iRFP 3T3 fibroblasts at the indicated time points. Cells were counted and plated at 2000 cells per well in 0.75% 1X DMEM soft agar onto a 1X DMEM 0.75% agarose layer. The white box indicates the area scanned in high resolution in (**C**) Scan settings: Resolution 169 µm, offset 4 mm, intensity (700 nm) L02. (**B**) Quantification of (**A**) at the indicated time-points. Graph represents the individual signal of replicate wells. Scan settings as in (**A**). (**C**) High resolution scans of colonies shown in (**A**) at indicated time-points. Scan settings: Resolution 21 µm, offset 4 mm, intensity (700 nm) L02. (**D**) Quantification of (**C**). Graphs represent growth analysis of individual colonies. Scan settings as (**C**).

Although colony formation assays are important for determining oncogenic transformation, anchorage independent growth assays (for example soft agar assays) correlate more closely with *in vivo* tumor growth of xenografts in mice [10]. To test if iRFP quantification can be applied to soft agar assays to measure anchorage independent growth, we plated parental iRFP expressing 3T3 cells (parental control) and cMyc/Ha-RasG12V infected iRFP 3T3 (transformed 3T3s) cells in double-layered soft agar. The wells were scanned and the iRFP signals were quantified approximately 30 minutes after plating to determine the starting level of fluorescence (Figure 2A, left panel). We scanned the plate repeatedly to monitor the growth behavior from individual cells into colonies over several days. As expected, the parental iRFP fibroblasts did not show any sign of growth by observation or by iRFP fluorescence quantification (Figure 2A right, top row), while the transformed iRFP 3T3s grew as iRFP positive colonies (Figure 2A right, bottom row). By quantifying the assays repeatedly over a period of 356 hours, we were able to generate growth curves of individual wells to visualize the increasing difference between the two cell populations over time (Figure 2B) with an observable difference between transformed to parental fibroblasts as early as day 6 (144 h, Figure 2B). To test if iRFP quantification can be used to monitor growth of individual soft agar colonies, high-resolution images of a population of transformed cells were scanned and the colony-growth behavior was documented over time (Figure 2C). Quantifying the signal of individual colonies allowed us to determine individual growth curves for each colony (Figure 2D). iRFP detection assay therefore is a powerful tool to monitor soft agar colony formation without outside intervention. This technique allows for the generation of growth curves on a per-well and a per-colony base over time, while at the same time retaining the sample for further analysis at the endpoint of the experiment.

**Figure 3:**
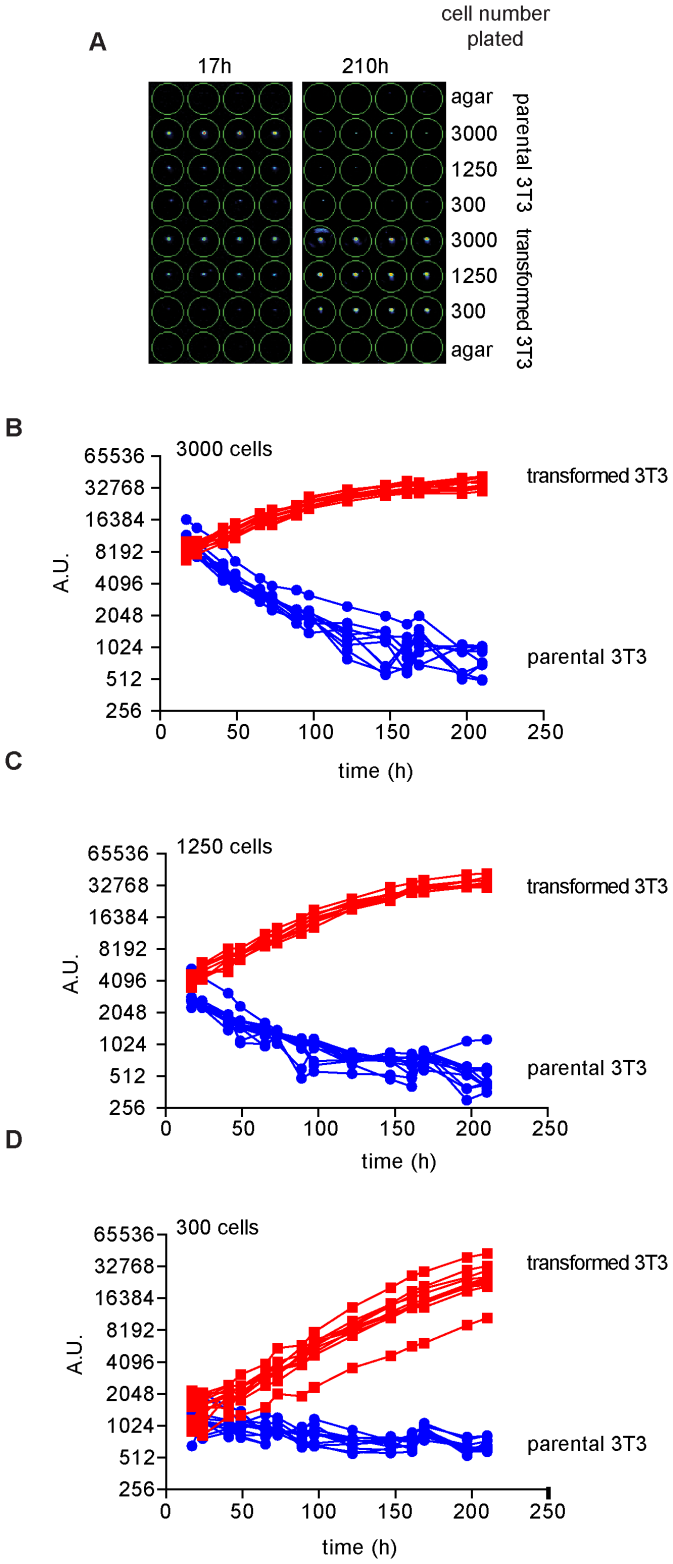
96 well based soft agar assay to determine anchorage independent growth kinetics with high throughput. (**A**) Odyssey LI-COR scans of representative cMyc/Ha-RasG12V infected or parental iRFP 3T3 fibroblasts at the indicated time points. Cells were counted and plated at 3000, 1250 or 300 cells per well onto a 1X DMEM 1.5% agarose layer. Scan settings: Resolution 169 µm, offset 4 mm, intensity (700 nm) L02. (**B-D**) Quantification of cells plated at 3000 (**B**), 1250 (**C**) and 300 (**D**) cells per well. Graphs represent growth analysis of individual wells. Scan settings as (**A**).

**Figure 4:**
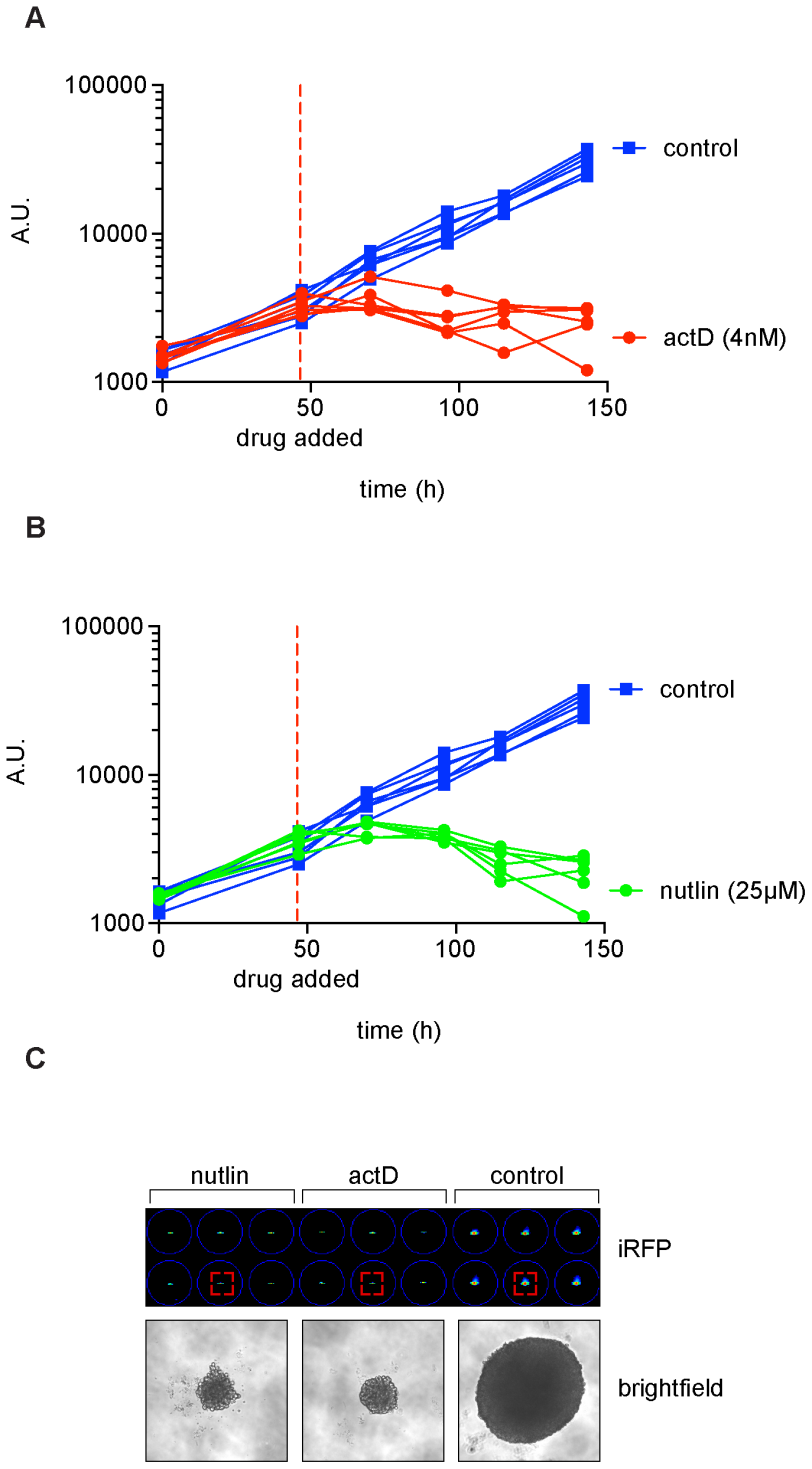
Effect of p53 inducing drugs on anchorage independent growth in high throughput soft agar assays. (**A,B**) cMyc/Ha-RasG12V infected 3T3 fibroblasts where plated at 550 cells per well onto 50 µl agarose. At the indicated time, nutlin (25 µM, **A**) or actinomycin D (actD, 4 nM, **B**) was added and colony growth monitored by repeated LI-COR scanning. Scan settings: Resolution 169 µm, offset 4 mm, intensity (700 nm) L02. (**C**) LI-COR scan and bright field images of colonies taken at the last time point of **A** and **B**. Red squares highlight the colony shown in the respective bright field image.

Classic double layer soft agar assays are not easily transferred into a high throughput setting, since once seeded between the agar layers the cells cannot be easily treated with compound or genetically modified with infection or transfection. To overcome these limitations we performed one layer soft agar assay, using the same cell pools in a 96 well based format, which can be easily used in a high throughput screening setup. In traditional soft agar assays, colonies arise from single cells. To replicate this in a 96 well assay, we plated transformed iRFP 3T3s at a very low concentration (approximately 0.7 cells in 150 µl) to achieve single cell per well cultures. Interestingly, there was a substantial amount of heterogeneity in the growth behavior of different cells (Figure S1A and B). This suggests that while single cells in single layer soft agar can be used to measure clonal anchorage independent growth, it may also generate heterogeneity and a high number of empty wells (Figure S1A and B). This is likely due to the low concentration of cells and the substantial impact on survival of each individual cell by slight variations of the baseline stress level in each individual wells during plating and at the early stages of the assay. In addition, the single cell approach shows a later onset and heterogeneity of colony outgrowth (Figure S1A and B). These complications do not pose a problem to relatively small-scale screens, where the operator could manually avoid treating wells with no cells. In unbiased high throughput applications such a setup would be unfavorable since a pre-screen of wells with cells will further complicate automation. Furthermore it would be favorable to eliminate the time it takes for the single cells to establish colonies. We therefore addressed these issues by starting with a higher cell number, which should reduce per well variability and speed up the process of colony formation. We plated cells in a single cell suspension at the indicated concentrations and quantified the iRFP signal at the indicated times (Figure 3A to D). The cells formed a single colony in the center of the well, comparable to colonies derived from single cells (compare Figure 3A and Figure S1B). This is presumably due to the curvature of the soft agar coating (Figure 3A). Over time the parental cells lost fluorescence, suggesting that the cells died, presumably due to lack of attachment. In contrast, transformed cells began to grow and increase in size over the duration of the experiment, irrespective of the initial cell number. Interestingly, the iRFP signal eventually reaches a similar level irrespective of the cell number plated. This is likely to be a combination of several effects including the high nutrient consumption of big colonies, which cannot be supplied by daily media change, as well as inadequate nutrient supply in the center of larger colonies. An observable difference between parental and transformed 3T3 cells seeded at the lowest density could be detected within two days (Figure 3D). This demonstrates that the simplified one layer soft agar assay in combination with iRFP quantification is a valid approach to determine soft agar based transformation, and the detection of transformation is much earlier and sensitive than traditional soft agar methods (2 to 3 days compared to over 1 week). This is of particular importance because transfections of screening libraries, due to their transient nature, rely on a relatively rapid evaluation. A further advantage of this system is that the cells do not have to be mixed with liquid agar but are seeded directly onto agar coated wells, which allows a much simpler format for robotization. In addition, cells can be easily transfected or infected with interfering RNAs, over-expression constructs or treated with compounds due to the lack of top agar layer. This study underscores the versatility of iRFP fluorescence based assay to determine colony formation and anchorage independent growth, which is invaluable for an unbiased search for therapeutic targets and for drug discovery. To test this, we seeded 550 transformed 3T3s onto 50 µl of soft agar. As observed before, these cells formed a colony in the middle of each well. At the indicated time the cells were treated with 25 µM nutlin, 4 nM actinomycin D (actD) or PBS (control) and the colonies monitored for growth behavior. As observed before, transformed control treated cells grew rapidly. In contrast to that, both nutlin and actimomycin D treatment inhibited colony growth compared to control cells (Figure 4A, B and C) and resulted in a gradual loss of iRFP signal. A clear difference between control and treated cells could be detected as early as 2 days after treatment. This shows that iRFP based 96 well soft agar assays are suitable for anchorage independent growth high content screening with drug induced growth inhibition as readout. In addition compounds that only affect the exposed layer of the colony or single cells would score relatively low. This increases the chance of identifying compounds with an *in vivo* activity, since solid tumors are unlikely to be treated with a compound that cannot inhibit the growth of a colony *in vitro*. Although the data shown is based on transformed mouse fibroblasts, the system can be easily adapted to a wide variety of cell lines and screening setups allowing a much quicker and unbiased quantification of both oncogenic transformation and its inhibition.

## Supporting Information

Figure S1 96 well based soft agar assay starting from single cells. (**A**) iRFP quantifications of single cell colonies at the indicated time points. Scan settings: Resolution 169 µm, offset 4 mm, intensity (700 nm) L02. (**B**) iRFP scans and bright field images of example colonies from single cells shown in (**A**) after 214 hours. Numbers represent colonies highlighted in (**A**).
(TIF)
