# Supplementary figures and images for: iRFP Is a Real Time Marker for Transformation Based Assays in High Content Screening
Source: PLoS One. 2014 Jun 2;9(6):e98399. doi: 10.1371/journal.pone.0098399 (PMC4041769; doi:10.1371/journal.pone.0098399)

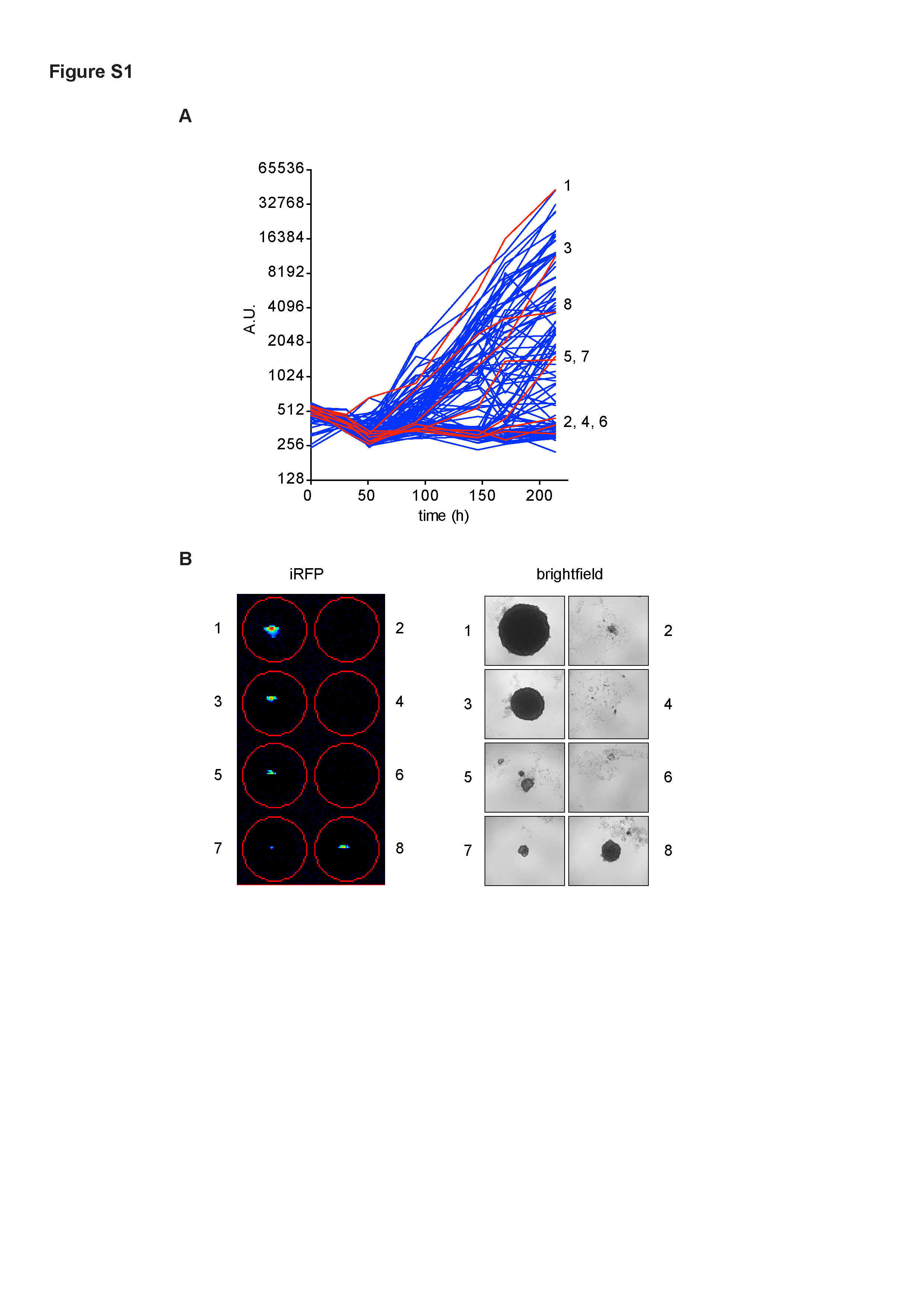

Supplement: Figure S1 — 96 well based soft agar assay starting from single cells. (A) iRFP quantifications of single cell colonies at the indicated time points. Scan settings: Resolution 169 µm, offset 4 mm, intensity (700 nm) L02. (B) iRFP scans and bright field images of example colonies from single cells shown in (A) after 214 hours. Numbers represent colonies highlighted in (A). (TIF) [file pone.0098399.s001.tif]
